# Profiling of the embryonic Atlantic halibut (*Hippoglossus hippoglossus* L.) transcriptome reveals maternal transcripts as potential markers of embryo quality

**DOI:** 10.1186/1471-2164-15-829

**Published:** 2014-09-30

**Authors:** Maren Mommens, Jorge MO Fernandes, Knut Erik Tollefsen, Ian A Johnston, Igor Babiak

**Affiliations:** Faculty of Biosciences and Aquaculture, University of Nordland, N-8049 Bodø, Norway; Aqua Gen AS, N-7462 Trondheim, Norway; Norwegian Institute for Water Research (NIVA), Gaustadalléen 21, N-0349 Oslo, Norway; School of Biology, Scottish Oceans Institute, East Sands, St. Andrews, Fife KY16 8LB UK

**Keywords:** Atlantic halibut, Egg quality, Embryonic development, Maternal transcripts, Microarray, Transcriptome

## Abstract

**Background:**

Commercial Atlantic halibut (*Hippoglossus hippoglossus)* farming is restricted by variable oocyte quality, slow growth, and early maturation of male fish. Maternally transferred components regulate early developmental processes; therefore, they have an effect on the future viability of the embryo. Using a newly developed Agilent 10 k custom-made oligonucleotide array, we profiled components of the transcriptome involved in immune defence as well as germline and muscle development during early developmental stages: 8-cell embryos (8CS), germ ring stage (GR), 10-somite stage (10SS), and hatched embryos (HT). In addition, we identified differentially expressed transcripts in low (≤9 ± 3% hatching) and high (≥86 ± 3°% hatching) quality eggs at 8CS to identify potential maternal markers for embryo quality.

**Results:**

Out of 2066 differentially expressed transcripts, 160 were identified as maternal transcripts being specifically expressed at 8CS only. Twenty transcripts were differentially expressed in 8-cell embryos between low and high quality egg groups. Several immune-related transcripts were identified as promising molecular markers of hatching success including *interferon regulatory factor 7* and *mhc class 2A chain*. Differential expression was positively validated with quantitative real-time PCR.

**Conclusions:**

We have demonstrated maternal transfer of innate and adaptive immune system transcripts into Atlantic halibut embryos and their relation with future embryo developmental potential. We identified several transcripts as potential molecular markers of embryo quality. The developed microarray represents a useful resource for improving the commercial production of Atlantic halibut.

**Electronic supplementary material:**

The online version of this article (doi:10.1186/1471-2164-15-829) contains supplementary material, which is available to authorized users.

## Background

Although the production of farmed Atlantic halibut (*Hippoglossus hippoglossus*) has increased over the last ten years it still faces significant bottlenecks. Gametes are obtained by hand-stripping and oocytes are frequently of variable quality because of non-optimal timing of gamete collection and stress to broodstock fish [1–3]. Compared to other farmed marine teleosts, Atlantic halibut embryos are poorly developed at hatching with an associated long yolk-sac resorption time and a requirement for extended feeding on live prey before they can be fed with commercial diets [4]. The long live feeding period makes the larvae vulnerable to bacterial and viral diseases and increases their mortality [5]. Gene expression studies of the Atlantic halibut immune system have so far focused on larvae and juveniles [6–9]. However, both innate and adaptive immune system-relevant factors are maternally transferred into teleost oocytes and present during embryonic development, before hatching [10]. Maternally transmitted immune factors have not yet been characterized in Atlantic halibut.

The relatively slow growth of Atlantic halibut juveniles and its sex-dependent growth dimorphism are the main obstacles to commercially viable Atlantic halibut production during the on-growing phase [11, 12]. The muscle growth characteristics of juvenile and adult stages of teleosts are moderated by environmental conditions at the embryonic stages, particularly temperature [13, 14]. Embryonic gene expression patterns of single myogenic regulatory factors, such as *myogenic differentiation 1* (*myod1*), *myogenic differentiation 2* (*myod2*), and *myogenin* (*myog*), as well as structural muscle proteins such as *myosin heavy chain* (*myhc*), *myosin light chain 2a* (*mylc2a*), and *myosin light chain 2b* (*mylc2b*), have been described in Atlantic halibut [15, 16].

All-female halibut populations are preferred in commercial farming because of its sex-dependent growth dimorphism. All-female Atlantic halibut production has been established in Canada and Norway [17, 18]. However, to ensure controlled reproduction, the underlying molecular mechanisms of gamete development in Atlantic halibut need to be better understood. For example, the present knowledge of genes controlling proliferation and migration of primordial germ cells (PGCs), precursors of gametes, is restricted in Atlantic halibut to embryonic expression of *askopos* (*kop*) and *Tudor domain-containing protein 5* (*tdrd5*) [19].

Early embryonic development in teleosts is driven until the start of zygotic transcription by maternally supplied mRNAs that are incorporated into the oocyte during oogenesis [20]. Maternal mRNAs are critical to embryonic development since they implement basic biosynthetic processes, direct first mitotic divisions, and specify initial cell fate and embryonic patterning [21]. Hence, maternal mRNAs are potential molecular markers for oocyte quality. In aquaculture, early estimation of oocyte quality can avoid costly and unnecessary incubation of low-quality material and improve production and predictability [22]. Several maternal mRNAs have been identified as molecular markers for oocyte quality in farmed mammals [23–26]. In commercially farmed teleosts, only *prohibitin 2 (phb2*) in rainbow trout (*Oncorhynchus mykiss*) has been found to be related to oocyte quality [27]. Previously, we have idenfied three uncharacterized maternal transcripts, correlating significantly with Atlantic halibut embryo quality [19]. In addition, we found indications that the transition from maternal to zygotic transcripts (MZT) in Atlantic halibut takes place between the blastula stage and germ ring stage [19, 28].

A microarray created by Douglas *et al*. [29] has been used to study gene expression in five developmental stages (hatched to post-metamorphosis) of Atlantic halibut, including larvae introduced to microencapsulated diet and juveniles fed fish meal replacement diets [30, 31]. Since the production of the first Atlantic halibut microarray [29], the number of Atlantic halibut ESTs available from NCBI GenBank (http://www.ncbi.nlm.nih.gov/dbEST) has increased by 40%, including 3670 maternal new ESTs [19, 32]. In the present study, we produced a new 10 k custom oligonucleotide array to profile embryonic expression of transcripts involved in immune system, germline, and muscle development, and to identify maternal transcripts. We also examined differentially expressed maternal transcripts in 8-cell embryos from batches with either high (≥86 ± 3%) or low hatching success (≤9 ± 3%) in order to identify potential molecular markers of embryo quality.

## Results

### Microarray transcript ontology

Of the 10279 probes designed and printed onto the microarray, 5364 (52%) represented transcripts with putative identification based on sequence similarity by BLASTX searches (cut-off E = 1E^−6^). GO annotations were obtained for 5009 (49%) probes. Most probes (61%) were classified as representing transcripts involved in cellular (24%), metabolic (23%), and regulatory processes (14%, Additional file 1). Their molecular function was dominated by binding (50%), catalytic activities (31%), and cellular compartments were cell (43%) and organelle (31%). A full list of GO annotation for the three domains is presented in Additional files 2, 3 and 4. After signal processing, data normalization, and filtering, 8533 (83%) probes were kept for further analysis of transcript expression during early embryonic development and 8447 (82%) for analysis of differentially expressed transcripts between high and low quality embryos at 8CS.

### Transcript expression during embryonic development

Among the four developmental stages, 2066 transcripts were differentially expressed and 339 (16%) were up-regulated only in one of the four developmental stages (*p* < 0.05). Out of the 339 up-regulated transcripts, 160 (47%) were strictly maternal transcripts, only expressed at 8CS, and 49 (12%), 54 (16%) 76 (22%) transcripts were only expressed at GR, 10SS and HT, respectively (Additional files 4, 5, 6, 7 and 8). No significantly enriched GO terms were found in any of the four groups of transcripts.

### Immune defence

**Figure 1:**
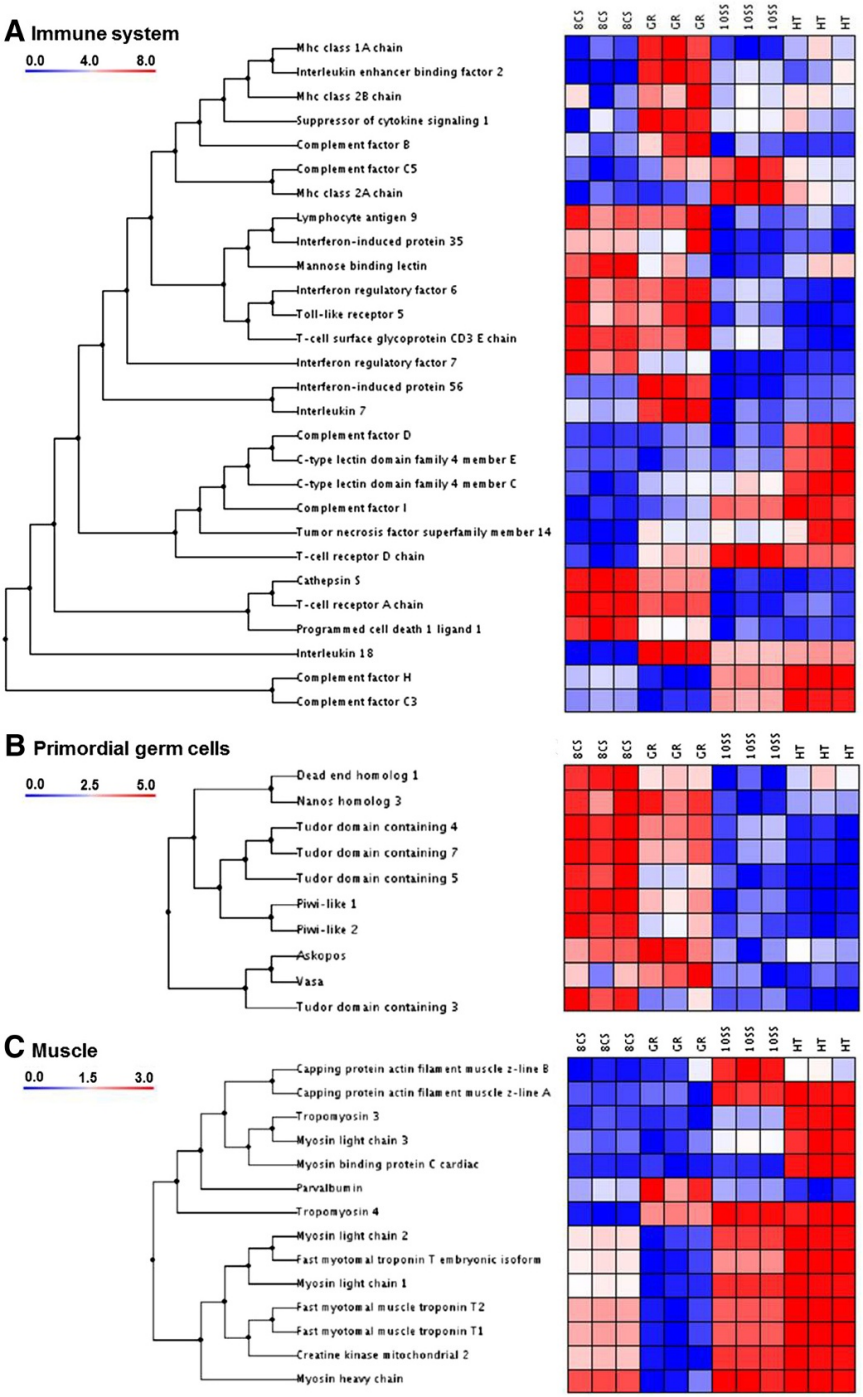
**Clustering of transcript groups. A**: Immune system, **B**: Germ cells, **C**: Muscle. Transcripts were clustered using the un-weighted pair-group method (UPGMA) using arithmetic averages with normal Euclidian distance as distance measurements. Developmental stages were: embryos at 8-cell stage (8CS); germ ring stage (GR), 10-somite stage (10SS), and hatched embryos (HT). Data were standardized against the first stage 8CS. Colour bar indicates relative expression in relation to 8CS. High intensity expression is represented in red colours while blue colours represent lower expression intensity (*n* = 3 batches).

Seven transcripts encoding complement system proteins were identified in Atlantic halibut embryos (Figure 1A). *Mannose binding lectin* (*mbl*) was highly expressed at 8CS, and to a lower degree at GR and HT. *Complement factor B* (*cfb*) was highly expressed at GR and *complement factor C5* (*c5*) at 10SS. *Complement factor C3* (*c3*), *H* (*cfh*) and *I* (*cfi*) were highly expressed at 10SS and HT. *Complement factor D* (*cfd*) was only expressed at HT. Two types of pattern recognition receptors (PRRs) were expressed: Toll-like receptors (TLRs), and C-type lectin receptors (CLRs). *Toll-like receptor 5* (*tlr5*) was highly expressed at 8CS and GR. *C-type lectin domain family 4 member C* (*clec4c*) was expressed at 10SS and HT while *C-type lectin domain family 4 member E* (*clec4e*) was only expressed at HT. Among transcripts encoding cytokines or related proteins, *interferon regulatory factor 7* (*irf7*) was expressed at 8CS and GR, *interferon-induced protein 35* (*ifi35*) at 8CS and *interferon regulatory factor 6* (*ifr6*) at GR and 10SS. *Interleukin enhancer binding factor 2* (*ilf2*), *interleukin 7* (*il7*), and *interferon-induced protein 56* (*ifi56*) were only expressed at GR. *Interleukin 18* (*il18*) was expressed from GR to HT. *Suppressor of cytokine signaling 1* (*socs1*) was highly expressed at GR.

Among the major histocompatibility complex (MHC) receptor sub-chain transcripts, *mhc class 1A chain* (*mhc1a*) and *mhc class 2B chain* (*mhc2b*) were highly present at GR while *mhc class 2A chain* (*mhc2a*) transcript level peaked at 10SS. Transcripts of the *T-cell receptor A chain* (*tcra*) and the *T-cell surface glycoprotein CD3 E chain* (*cd3e*) were found at 8CS and GR while *T-cell receptor D chain* (*tcrd*) was expressed from GR to HT. Three transcripts coding T-cell co-signaling regulators were identified: *Lymphocyte antigen 9* (*ly9*), expressed at 8CS and GR, *tumor necrosis factor superfamily member 14* (*tnfsf14*), expressed from GR to HT, and *programmed cell death 1 ligand 1* (*pd-1 l)* highly expressed at 8CS. *Cathepsin S* (*ctss*) was expressed at 8CS and GR.

### Primordial germ cells

All selected transcripts involved in primordial germ cell proliferation and migration were found at both 8CS and GR, except for *tudor domain containing 3* (*tdrd3*) being only expressed at 8CS (Figure 1B). Transcript level of *tudor domain containing 4* (*tdrd4*), *tudor domain containing 5* (*tdrd5*), *tudor domain containing 7* (*tdrd7*), *nanos homolog 3* (*nanos3*), *dead-end homolog 1* (*dnd1*), *piwi-like 1* (*piwil1*) and *piwi-like 2* (*piwil2*) was highest at 8CS, while the level of *vasa* (*vasa*) and *askopos* (*kop*) peaked at GR.

### Muscle development

*Myosin light chain 1* (*mylc1*) and *2* (*mylc2*), *myosin heavy chain* (*myh*), *fast myotomal troponin T embryonic isoform* (*eftnt*), *fast myotomal muscle troponin T1* (*ftnt1*) and *T2* (*ftntf2*) and *creatine kinase mitochondrial 2* (*ckmt2*) were expressed at 8CS, and increasingly at 10SS and HT (Figure 1C). *Capping protein actin filament muscle z-line A* (*capza*) was highly expressed at 10SS and HT, and *Capping protein actin filament muscle z-line B* (*capzb*) was predominantly expressed at 10SS. *Tropomyosin 3* (*tpm3*), *myosin light chain 3* (*mylc3*), and *myosin binding protein C cardiac* (*mybpc3*) were highly expressed at HT. *Parvalbumin* (*pval*) was highly expressed at GR and *Tropomyosin 4* (*tpm4*) at GR, 10SS, and HT.

### Differential transcript expression in high and low quality eggs

**Figure 2:**
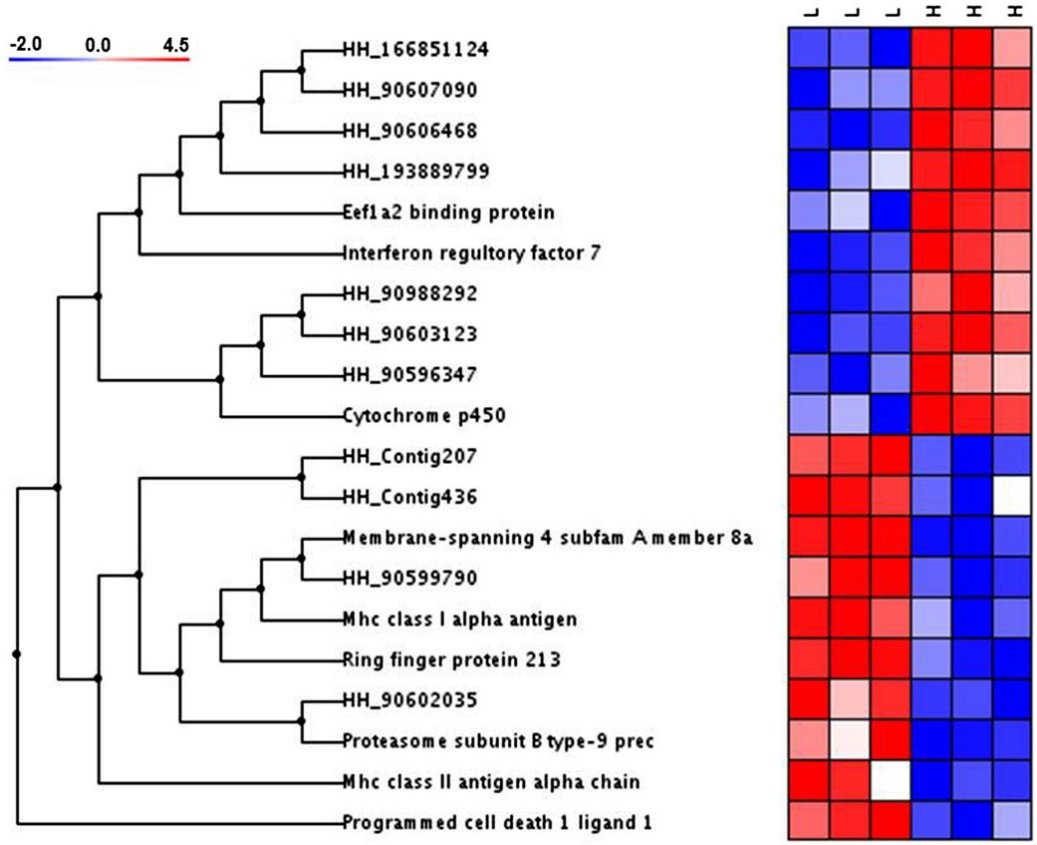
**Clustering of differentially expressed transcripts in high (H) and low (L) quality oocytes.** Transcripts were clustered using the un-weighted pair-group method (UPGMA) using arithmetic averages with normal Euclidian distance as distance measurement. High intensity expression is represented in red colours while blue colours represent lower expression intensity (*n* = 3 batches).

**Figure 3:**
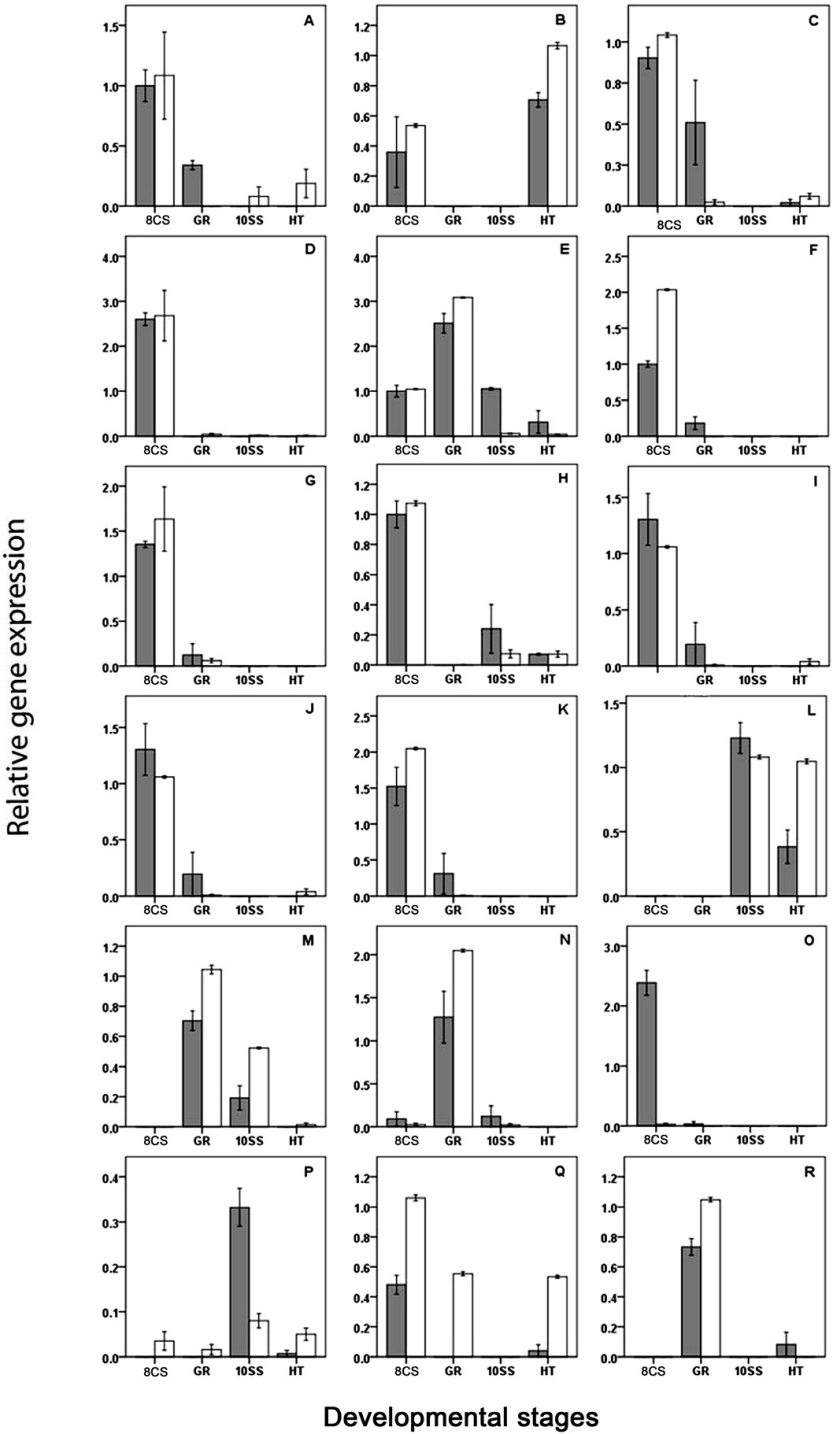
**Validation of expression patterns by quantitative PCR (qPCR).** Relative gene expression (±SD) in Atlantic halibut embryonic developmental stages estimated by microarray (grey bars; *n* = 3) and qPCR (white bars; *n* = 5). The embryonic developmental stages (*n* = 3) were: 8-cell stage (8CS), germ ring (GR), 10-somite stage (10SS), and hatched larvae (HT). qPCR data were normalized with *Luc*. Data were standardized against the 8CS stage. **A**: *irf7*, **B**: 90603123, **C**: HH_90988292, **D**: *eef1a2bp*, **E**: *cyp2n*, **F**: HH_193889799, **G**: HH_90607090, **H**: HH_166851124, **I**: HH_90606468, **J**: *rnf213*, **K**: HH_Contig436, **L**: *psmb9*, **M**: HH_90602035, **N**: HH_90599790, **O**: *pd-1 l*, **P**: *ms4a8a*, **Q**: HH_Contig207, and **R**: *mhc1a*.

Twenty transcripts were differentially expressed between high and low quality 8CS embryos (*p* < 0.05; Figure 2 and Table 1). Gene set enrichment analysis resulted in no significant differences in functional terms. The highest differences in transcript levels were found for *irf7* (8.0 ± 0.3-fold lower in low quality 8CS embryos, Table 1) and *mhc2A* (7.8 ± 0.7-fold higher in low quality 8CS embryos). Nine transcripts (57%) had significant BLAST hits representing genes involved in immune response, metabolism, RNA transcription, protein degradation, cell signalling, and cytoskeleton. Conserved domain searches in the remaining 8 differentially expressed transcripts without significant BLAST hits resulted in identification of HH_90606468 as belonging to the chaperonin-like superfamily of proteins. Two differentially expressed transcripts, *eef1a2bp* and *pd-l1* showed strictly maternal expression and were expressed only before GR (Figure 3 and Additional file 5).

**Table 1:** **Differentially expressed transcripts in high and low quality Atlantic halibut 8CS embryos**

				Microarray		qPCR	
Function	Probe name	GeneBank Accession	BLAST hit gene description ( ***Abbreviation*** )	Fold-change (±SD)	Adj. ***p*** -value	Fold-change (±SD)	***p*** -value
Immune response	HH_90607192	EB040633	*interferon regulatory factor 7* (*irf7*)	↓8.0 ± 0.3	7E-06	↓8.9 ± 0.0	0.005
Uncharacterized	HH_90603123	EB036564	NA	↓7.8 ± 0.2	2E-06	↓8.3 ± 0.0	< 0.001
Uncharacterized	HH_90988292	EB103461	NA	↓6.4 ± 0.1	4E-05	↓6.8 ± 0.2	0.005
Uncharacterized	HH_90596347	EB029788	NA	↓5.8 ± 0.5	4E-04	↓6.4 ± 0.3	< 0.001
RNA transcription	HH_90604682	EB038123	*eef1a2 binding protein* (*eef1a2bp*)	↓3.8 ± 0.1	2E-04	↓4.7 ± 0.2	0.005
Metabolism	HH_90596960	EB030401	*cytochrome p450* (*cyp2n*)	↓3.5 ± 0.1	1E-04	↓3.8 ± 0.5	0.004
Uncharacterized	HH_193889799	FK703165	NA	↓3.4 ± 0.6	2E-04	↓4.2 ± 0.4	0.037
Cytoskeleton	HH_90607090	EB040531	microtubule-associated protein homolog	↓2.7 ± 0.3	1E-04	↓2.4 ± 0.6	0.021
Uncharacterized	HH_166851124	FD698747	NA	↓2.7 ± 0.5	2E-04	↓2.5 ± 0.7	< 0.001
Uncharacterized	HH_90606468	EB039909	unnamed protein product [*Tetraodon nigroviridis*]	↓2.2 ± 0.4	3E-04	↓3.1 ± 0.2	0.001
Immune response	HH_38317730	EB173954	*mhc class ii antigen alpha chain* (*mhc2a*)	↑7.8 ± 0.7	3E-04	↑6.1 ± 0.1	< 0.001
Protein degradation	HH_90598492	EB031933	*ring finger protein 213* (*rnf213*)	↑4.8 ± 0.5	8E-06	↑5.2 ± 0.3	< 0.001
Uncharacterized	HH_Contig436	EB036359	chromosome 3 open reading frame 17 protein	↑3.9 ± 0.3	3E-04	↑4.2 ± 0.0	0.002
Protein degradation	HH_Contig1979	EB036110	*proteasome subunit beta type-9 precursor* (*psmb9*)	↑3.7 ± 0.6	4E-04	↑3.8 ± 0.1	0.001
Uncharacterized	HH_90602035	EB035476	NA	↑3.3 ± 0.4	1E-04	↑3.0 ± 0.1	0.019
Uncharacterized	HH_90599790	EB033231	NA	↑3.2 ± 0.2	8E-05	↑3.7 ± 0.1	< 0.001
Immune response	HH_Contig2081	EB031478	*programmed cell death 1 ligand 1* (*pd-1 l*)	↑3.0 ± 0.3	1E-04	↑2.8 ± 0.1	0.003
Cell signalling	HH_Contig1463	EB040282	*membrane-spanning 4 subfamily a member 8a* (*ms4a8a*)	↑2.9 ± 0.4	2E-05	↑2.5 ± 0.2	< 0.001
Uncharacterized	HH_Contig207	EB041560	NA	↑2.7 ± 0.1	6E-05	↑2.1 ± 0.3	< 0.001
Immune response	HH_Contig2306	EB036581	*mhc class i alpha antigen* (*mhc1a*)	↑2.5 ± 0.2	3E-04	↑3.4 ± 0.0	0.013

For each transcript, fold-change difference (±SD) in low quality oocytes estimated by microarray (*n* = 3) and qPCR (*n* = 8) is given. Transcripts are grouped according to their function.

### Microarray validation

A significant correlation was found between qPCR and microarray results (ϱ = 0.88, *p* < 0.0001, *n* = 276; Additional file 9). Significant differences in transcript levels between high and low quality 8CS embryos by microarray were confirmed by qPCR for all 20 differentially expressed transcripts (Table 1). Expression patterns of the differentially expressed transcripts during embryonic development (*n* = 18) followed the same general trend when estimated by qPCR, with a few exceptions. Microarray relative expression data revealed strictly maternal expression (no expression at GR, 10SS and HT) of two transcripts (Figure 3D and O) while according to qPCR data, four transcripts were strictly maternal (Figure 3D, F, K, and O). *Membrane-spanning 4-domains subfamily a member 8a* (*ms4a8a*) was expressed only zygotically (no expression before stage 10SS) according to microarray results, but showed maternal-zygotic expression (expressed at all four developmental stages) according to qPCR expression results (Figure 3P). HH_Contig207 expression was detected at 8CS and HT by microarray and addition in GR by qPCR (Figure 3Q). *Mhc class 1A chain* (*mhc1A*) expression was apparent in GR and HT by microarray, but only in GR by qPCR (Figure 3R). For 14 out of 20 tested transcripts, qPCR expression levels were up to 1.5 times higher in one or more developmental stages, compared to the microarray results (Figure 3).

## Discussion

In hatched Atlantic halibut embryos, the anterior part of the head kidney is present, but no haematopoietic tissue or cells can be observed. Liver, thymus, and spleen, important for the development of the adaptive immune system, are not present yet [7]. Therefore, the innate immune system is the first line of immune protection. The complement system is a major non-cellular component of the innate immune system [33]. The presence of maternally transferred *mbl* transcripts, an activator of the lectin pathway (LP), indicates that it is the first complement defense mechanism active in Atlantic halibut embryos (Figure 1A). Between GR and HT, expression of transcripts encoding central complement components (*c3* and *c5*) and transcripts encoding alternative pathway (AP) regulatory proteins (*cfb*, *cfd*, *cfh*, and *cfi*) increases. As previously found in zebrafish and rainbow trout, the AP seems to be functional as immune defense in Atlantic halibut embryos, before the adaptive system is developed [34, 35]. Despite the lack of IgM-bearing cells and organs of the adaptive immune system in Atlantic halibut embryos, transcripts of MHC receptors (*mhc1a*, *mhc2a*, *mhc2b*), T-cell receptors (TCR, *tcra*, *tcrd*), and T-cell co-signaling regulators (*ly9*, *tnfsf14*, *pd-1 l*, *ctss*) were present. These maternally transferred transcripts of the adaptive immune system add to the immune protection of the developing embryo and larvae. A similar transfer of maternal transcripts involved in the innate and adaptive immune system have been identified in in half-smooth tongue sole (*Cyngolossus semilaevis*) and rainbow trout embryos [36, 37].

Transcripts of three *tdrd* genes, *tdrd4*, *tdrd5* and *tdrd7*, showed similar expression pattern as *piwil1* and *piwil2*, reflecting the close interaction of the corresponding protein products (Figure 1B). TDRD4, TDRD5, and TDRD7, together with TDRD1, TDRD6, TDRD8 and TDRD9 form PIWI-TDRD complexes that are essential in retrotransposon silencing, chromatoid body assembly and spermiogenesis [38]. *Tdrd5* maternal expression has previously been identified in Atlantic halibut embryos [19]. Among *tdrd* transcripts, *tdrd3* was the only transcript expressed mainly at 8CS. TDRD3 preferably binds to asymmetric dimethyl arginine marks (aDMAs) in somatic cells acting as a transcriptional co-activator [39]. Expression profiles of *nanos3* and *dnd1* clustered together with *piwil* and *tdrd* transcripts with the exception of increased expression at H. *Dnd1* and *nanos3* code germ plasm and PGCs specific RNA-binding proteins involved in differentiation and survival of PGCs [40, 41]. Through binding to 3’UTRs of target mRNAs, DND1 counteracts miRNA-mediated posttranscriptional repression in PGCs [40, 42]. Expression of *dnd1* and *nanos3* in Atlantic halibut embryos was similar to expression patterns found in teleost embryos previously [40, 43, 44]. Expression of *kop* and *vasa* peaked at GR in Atlantic halibut, diminishing during later embryonic development and at HT. A similar expression pattern of *kop*, coding a PGC-specific P-loop protein of unknown function, has previously been identified in zebrafish and Atlantic halibut [19, 45]. *Vasa*, an ATP-dependent RNA helicase, is mostly known as a PGC marker, but has recently also been identified as a regulator of cell cycle progression in somatic cells [46, 47].

Most muscle related transcripts identified during Atlantic halibut embryonic development represented isoforms of transcripts coding structural muscle proteins members of myosin (*myh*, *mylc1*, *mylc2*, *mylc6*, and *mycl3*), troponin (*ftnt1*, *ftnt2*, and *eftnt*), tropomyosin (*tpm3* and *tpm4*) and parvalbumin (*pval*; Figure 1C). Myosin molecules consist of six subunits, two heavy chains (MYHs) of ~200 kDa and four light chains (MYLs) of ~20 kDa. *Myhc* expression has previously been identified at the 17-somite stage and onwards in Atlantic halibut [15]. In the same study, two isoforms of *mylc2* were identified to be stage-specific (embryonic: *mylc2a* and larval/juvenile: *myl2b*). In zebrafish, *myl1* and *myl2* are expressed at the 10-somite stage, followed by *myl3* expression at the 12-somite stage. Here we have identified isoforms of *myl1* and *myl2* with expression at 8CS, 10SS, and HT, while *myl3* was mainly expressed at HT. One embryonic/larval troponin isoform (*efTnThh*) and two adult isoforms have previously been identified (*efTnThh-1* and *efTnThh-2*) in Atlantic halibut larvae during metamorphosis [48]. In the present study, *ftnt1*, *ftnt2* and *eftnt* showed similar expression patterns, indicating that they represented embryonic/larval isoforms.

In Atlantic halibut embryos, *tpm3* expression started after somite formation (10SS) and was highly expressed at HT. Isoforms of *tpm3* have been previously found to be essential for embryonic development in mice, but the underlying mechanism is unknown [49]. Both *tpm4* (also named δ*tpm*) and *mybpc3* are known to be specifically expressed in cardiac muscle in zebrafish [50, 51]. While *tpm4* expression was detected from GR to HT, *mybpc3* expression was restricted to HT. In the present study, *pval* expression peaked earlier, at GR, in Atlantic halibut embryos compared to its expression in zebrafish, where it was first detected in 15-somite stage embryos [52]. As a Ca^2+^-binding protein, PVAL is usually present in high concentrations in fast muscle cells, to a lower extend in specific neurons of the central and peripheral nervous system, and in cells of endocrine glands [53].

The two maternal transcripts, *irf7* and *mhc2a*, were identified as potential markers for quality in Atlantic halibut due to their high level of expression differences in high and low quality 8CS embryos (Table 1). The three uncharacterized transcripts (HH_90603123, HH_90988292, and HH_90596347) had the same potential, but their functions have to be further characterized. IRF7 is the primary regulator of type I interferon (IFN) production and its absence impairs antiviral innate immunity [54]. The significantly lower levels of *irf7* transcripts in low quality 8CS embryos may result in reduced immune response capacity resulting in further poor embryonic and larval development. Most of the transcripts showing elevated expression in low quality embryos are involved in adaptive immune response and protein degradation (Table 1). Their elevated concentrations could be the result of a maternally transferred immune response during the oogenesis triggered by unknown inflammation, infection or immune-activated stress.

Polyadenylation of maternal mRNAs during oocyte maturation usually protects mRNAs from degradation and activates their translation [55]. In contrast, regulatory RNA or protein-mediated deadenylation triggers mRNA degradation and translational repression to allow normal embryonic development after the maternal-zygotic transition (MZT) [56]. In *Xenopus tropicalis*, oocyte post-ovulatory aging (POA) induced a general decrease in maternal transcripts and a female-specific shortening of maternal mRNAs by deadenylation in oocytes that developed into embryos experiencing high malformation and mortality rates [57]. In contrast, POA induced both a decrease and increase in specific maternal transcripts in rainbow trout oocytes [58, 59]. In the present study, timing of hand-stripping Atlantic halibut females was synchronised to their individual ovulation rhythms to avoid POA. Whether low maternal transcript levels are the result of a lack or sub-optimal polyadenylation during Atlantic halibut oocyte maturation, leading to poor transcript translation and/or degradation in low quality early embryos, requires further investigation. Due to the experimental design, it was not possible to estimate whether the transcript level differences in high and low quality 8CS embryos were based on individual female differences and/or inheritability. However, no major differences in transcript levels were observed within the three oocyte batches tested (Figure 2). To test the potential of *irf7* and *mhc2a* as markers for Atlantic halibut oocyte quality, future studies should nevertheless be performed across a higher number of batches from females of known genetic background.

Both abundance of differentially expressed transcripts in high and low quality oocytes (Table 1) and their expression patterns during embryonic development (Figure 3) were successfully confirmed by qPCR. The qPCR and microarray analysis have inherent technical and data normalization challenges that can lead to variability in results. Following strict quality assessment procedures in both techniques, (RNA quality, cross-hybridisation of microarray probes, microarray spot intensity, qPCR primer design, and PCR efficiency) and data filtering after normalization (cut-offs for fold-changes and low microarray spot intensity) resulted in high correlation between qPCR and microarray data (Additional file 9) [60, 61]. Due to the requirement of designing qPCR primers across exon/introns boundaries, microarray probes and qPCR primer locations varied. This could result in the observed variation between qPCR and microarray data. Compared to the previously created Atlantic halibut microarray by Douglas et al. [29], the new microarray contains a higher number of transcripts corresponding to genes expressed during the embryonic development. This microarray was successfully used to screen maternal transcript expression, and to identify differential transcripts expression in low and high quality 8CS embryos. It has proven to be suitable for future analysis of Atlantic halibut embryonic transcript expression which is likely to advance our understanding of important developmental processes in teleosts.

## Conclusions

Using a new Atlantic halibut 10 k custom oligonucleotide array, we have demonstrated maternal transfer of innate and adaptive immune system transcripts into Atlantic halibut embryos and profiled their expression in early developmental stages. We identified several transcripts, including *irf7* and *mhc2a*, as potential molecular markers for embryo quality. Microarray validation did prove the usefulness of the tool for further transcript quantification in Atlantic halibut. Both the established information and microarray provide useful resources to improve commercial production of Atlantic halibut.

## Methods

### Fish husbandry and sample collection

All procedures of fish husbandry and sample collection were in accordance with the guidelines set by the National Animal Research Authority (Forsøksdyrutvalget, Norway). For transcript expression profiling during early development, embryos were collected at the 8-cell stage (8CS), 8 h post fertilisation (hpf); germ ring stage (GR), 82 hpf; 10-somite stage (10SS) 142 hpf; and hatched larvae (HT), 340 hpf, from eight females (30–40 kg) at a commercial Atlantic halibut farm (Risørfisk AS, Risør, Norway). All oocytes were fertilized *in vitro* with pooled sperm from two random males (15–20 kg) at the peak of their reproductive season. Females and males were fed EWOS Premix (EWOS, Bergen, Norway) and kept under natural photoperiod conditions. Eggs were incubated in large-scale 280 L incubators at salinity 34 ± 1 ‰ and temperature 6.3 ± 0.1°C. Fertilization and hatching percentage was estimated at 8CS and 340 hpf, respectively. Sampling was performed at 8CS to ensure that only fertilized oocytes were collected. Samples were immediately snap-frozen in liquid nitrogen. Only high quality embryos defined as fertilization success ≥ 90 ± 2% and hatching success ≥ 85 ± 2% were used for studies of normal development. Three separate batches of eggs were used for these experiments.

To identify differentially expressed transcripts between high and low quality 8CS embryos, oocytes were collected at the University of Nordland (Bodø, Norway) from 20 females (40–60 kg) kept under natural photoperiod conditions and fed Fish Breed-M (INVE Aquaculture NV, Dendermonde, Belgium). All oocytes were fertilized *in vitro* with sperm pooled from two random males. Eggs were incubated in 100 × 15 mm Petri dishes in triplicates, approximately 100 eggs per dish, at 5.5 ± 0.5°C in 33 ± 1 ‰ filtered seawater, added 0.5% (v/v) penicillin-streptomycin-neomycin solution (5000 U penicillin, 5 mg streptomycin, and 10 mg neomycin per ml, Sigma, St. Louis, Mo, USA) until hatching at 340 hpf. Samples were snap-frozen in liquid nitrogen. Fertilization and hatching percentages were estimated as described above. Fertilization and hatching percentage was used to categorize collected embryo groups as high and low quality embryos. Embryos with low fertilization rate (≤16 ± 3%) and low hatching percentage (≤7 ± 3%) were defined as low quality embryos (L) and embryos with high fertilization (≥91 ± 2%) and high hatching percentage (≥86 ± 3%) were defined as high quality embryos (H). Three groups of high and three groups of low quality embryos were selected to identify differentially expressed maternal transcripts at the 8CS stage.

### Microarray construction and probe design

Approximately 22,000 ESTs were obtained from the NCBI GenBank and subjected to EST pre-processing, clustering and contig assembly using a local installation of ESTExplorer (http://estexplorer.biolinfo.org). In essence, vectors were removed, low quality sequence repeats were masked, and the resulting sequences subjected to clustering and contig assembly using semi-rigid parameters (CAP3 = 80%, 50 ORFs). The resulting 3,105 consensus sequences (contigs) and 7,174 single ESTs (singletons) were subjected to blasting, mapping and annotation by a local installation of Blast2GO (http://www.blast2go.org/) using default parameters with minor modifications. In short, sequences were blasted against the NCBI non-redundant database using BLASTX (E = 10E-3). These results were complemented with a BLASTX against UniProtKB/Swiss-Prot (http://www.ebi.ac.uk/uniprot). Sequences with blast hits were then mapped against the Blast2Go database and resulting mapped sequences annotated in a sequential manner according to decreasing cut-off values (1: E = 10E-6, cut-off: 55, HSP coverage cut-off: 75; 2: E = 10E-6, cut-off: 55, HSP coverage cut-off: 0 and 3: E = 10E-6, cut-off: 60, HSP coverage cut-off: 0, Evidence code weight: ISS = 1.0, IEA = 1.0). Gene ontology (GO) results were enriched by merging Interpro (http://www.ebi.ac.uk/interpro/) annotations to existing GOs as well as GOs augmented by the Blast2Go functionality ANNEX. GO distributions of array probes were displayed after reducing GO complexity using GOslim (generic) [62]. Redundant sequences were removed on the basis of sequence similarity (>70% similarity) and array probe cross-hybridization potential. The resulting 10,279 sequences were subjected to 60-mer probe production by eArray (https://earray.chem.agilent.com/earray/). Probes were printed in quadruples on a 4x44k Agilent custom oligoarray (Agilent Technologies, Santa Clara, US).

### RNA extraction

Total RNA from each developmental stage (8CS, GR, 10SS, and HT; *n* = 3 batches each) and quality type of 8CS embryo (L, H; *n* = 3 batches each) was extracted according to the Tri reagent method (Sigma, St-Louis, MO, USA) using QIAazol (Qiagen, Nydalen, Sweden). RNA quality was initially checked by gel electrophoresis on a 1% (v/w) agarose gel containing SYBR safe™ DNA gel stain (Invitrogen, Paisley, UK). Genomic DNA was removed by DNAse treatment by Ambion Turbo DNA-free kit (Applied Biosystems, Austin, TX, USA). RNA quality was controlled by photometric analyses (260/230 > 1.8, 260/280 > 1.5) using NanoDrop spectrophotometer (Nanodrop Technologies, Wilmington, DE, USA). RNA integrity and quality was then estimated on Agilent 2100 Bioanalyzer and RNA integrity number (RIN) index was calculated for each sample using the Agilent 2100 Expert software. RIN provides a numerical assessment of the integrity of RNA that facilitates the standardization of the quality interpretation; for microarray processing, only RNAs with RIN number > 9.0 were further processed to reduce experimental biases due to poor RNA quality.

### Sample labelling and hybridization

For each sample, total RNA (200 ng μl^−1^) was labelled and amplified with Cy3-dCTP in duplicate using the Agilent Low Input Quick Amp Labelling kit according to the manufacturer’s protocol. Samples were spiked with Agilent One-Color Spike-Mix (1:10). The labelled and amplified cRNA was purified using the Qiagen RNease mini spin kit. Cleaned cRNA was quantified using a NanoDrop spectrophotometer. All samples had cRNA yields > 1.65 and Cy3 specific activity > 6.0. For each developmental stage (8CS, GR, 10SS and HT) and quality type of 8 CS embryo (L, H) three replicates were hybridized at 65°C for 17 h. After hybridization, all arrays were washed according to manufacturer’s protocol followed by a final acetonitrile wash. Slides were immediately scanned using an Agilent High density microarray scanner at 5 μm resolution (Agilent Technologies).

### Microarray validation by quantitative real-time PCR

To confirm expression results obtained from microarray analysis, primers were designed for 20 transcripts that were found to be differentially expressed between high and low quality 8CS embryos (Additional file 10). Total RNA was extracted from early embryos at 8CS, GR, 10SS, and HT (*n* = 5) and low (*n* = 8) and high (*n* = 8) quality 8CS embryos as described above and cDNA synthesized using QuantiTect Reverse Transcription kit (Qiagen). Primers were selected close to the 3’ end of the respective EST’s for each transcript. Whenever possible, primers were designed to cross at least one intron/exon border containing both donor and acceptor sites, in order to avoid amplification of any contaminating genomic DNA. Primer pairs for qPCR amplification were designed manually and screened for hairpins, homo- and cross-dimers using *Netprimer* (http://www.premierbiosoft.com/netprimer/). qPCR was performed as described in Fernandes et al. [28]. *Luciferase* (*Luc*, Promega, Madison, WI, US) was used as an external reference to normalize relative gene expression during embryonic development. β*-Actin* (*Actb*) and β*2-tubulin* (*Tubb2*) were used as reference genes to normalize relative gene expression between high and low quality 8 CS embryos (Additional file 11).

### Data analysis

Scanned images were analysed with the Agilent feature extraction Software Version 7.2. Resulting raw data were normalized (75 Quantile, median to baseline of all samples). Features were filtered based on their signal intensity values by satisfying the upper and lower percentile cut-offs 20–100% and outliers were removed with GeneSpring GX 10.0.2 (Agilent Technologies). All data were filtered for missing values and replaced by row mean imputation. A cut-off of ≥ 0.5 was applied to all data in addition to standard background correction to remove features close to the mean low intensity threshold across all arrays (0.38 ± 0.19). Expression analysis, functional profiling and hierarchical clustering were performed using the Babelomics 4.3 analysis suite (http://babelomics.bioinfo.cipf.es). Differentially expressed transcripts in the four embryonic developmental stages and between low and high quality 8CS embryos were estimated using *limma* with Benjamin and Hochberg false discovery rate (FDR) multiple-test correction (*p* < 0.05) [63, 64] and a 2-fold-change cut-off. Differentially expressed transcripts during developmental stages were filtered for transcripts up-regulated in only one of the developmental stages. Hierarchical clustering (un-weighted pair-group method with arithmetic averages (UPGMA) with normal Euclidian distance as distance measurement) was performed on selected transcripts involved in immune defence, PGC development, and muscle development among the filtered transcripts up-regulated in only one of the developmental stages. The same hieratchical clustering was performed for all differentially expressed transcripts between low and high quality 8CS embryos. Gene Ontology (GO) term enrichment using FatiGO + (Fishers exact test, two-tailed, Adj. *p* < 0.05) [65] was performed for transcripts up-regulated in one of the four embryonic stages and differentially expressed in low and high quality oocytes. For microarray validation, qPCR data were log_2_ transformed to be comparable with the microarray results. Correlation between qPCR and microarray data was estimated by Spearman’s Rho (ϱ). Mann–Whitney *U* (*p* < 0.05) was used to determine significant fold-change differences in relative gene expression between high and low quality 8CS embryos obtained by qPCR.

The microarray data have been deposited in the National Center for Biotechnology Information GEO (NCBI GEO; http://www.ncbi.nlm.nih.gov/geo) and are accessible under the GEO series accession number GSE61051.

## Electronic supplementary material

Additional file 1: **GO annotations for 10 k Atlantic halibut microarray.** A: Biological processes, B: Molecular functions, C: Cell components. (DOCX 90 KB)

Additional file 2: **Biological process (BP) gene ontology annotations (GOs) for Atlantic halibut 10 k microarray probes.** (DOCX 19 KB)

Additional file 3: **Molecular function (MF) gene ontology annotations (GOs) for Atlantic halibut 10 k microarray probes.** (DOCX 16 KB)

Additional file 4: **Cellular component (CC) gene ontology annotations (GOs) for Atlantic halibut 10 k microarray probes.** (DOCX 16 KB)

Additional file 5: **Up-regulated transcripts at 8CS.** (XLSX 27 KB)

Additional file 6: **Up-regulated transcripts at GR.** (XLSX 14 KB)

Additional file 7: **Up-regulated transcripts at 10SS.** (XLSX 14 KB)

Additional file 8: **Up-regulated transcripts at HT.** (XLSX 17 KB)

Additional file 9: **Microarray validation.** Correlation plot of fold-change differences from 20 differentially expressed genes analyses by microarray and qPCR (*n* = 276). Correlation is given as Spearman’s Rho (ϱ). Abbreviations: H: High quality oocytes, L: Low quality oocytes; 8CS: 8-cell stage, GR: Germ ring, 10SS: 10-somite stage and HT: Hatched embryo. (DOCX 327 KB)

Additional file 10: **Primer information for microarray validation.** (DOCX 18 KB)

Additional file 11: **Raw cycle threshold (CT) levels (mean ± S.E.) of reference genes for qPCR normalization.** A) *Act*β and B) *Tubb2* in high (H) and low (L) quality Atlantic halibut oocytes (*n* = 8). C) *Luc* during early embryonic development of Atlantic halibut (*n* = 5). 8CS: 8-cell stage; GR: germ ring stage, 10SS: 10-somite stage, and HT: hatched embryo. (DOCX 41 KB)
