# Supplementary material for: Profiling of the embryonic Atlantic halibut (Hippoglossus hippoglossus L.) transcriptome reveals maternal transcripts as potential markers of embryo quality
Source: BMC Genomics. 2014 Sep 30;15(1):829. doi: 10.1186/1471-2164-15-829 (PMC4246526; doi:10.1186/1471-2164-15-829)

Additional File - 1

GO annotations for 10k Atlantic halibut microarray.

A: Biological processes, B: Molecular functions, C: Cell components.


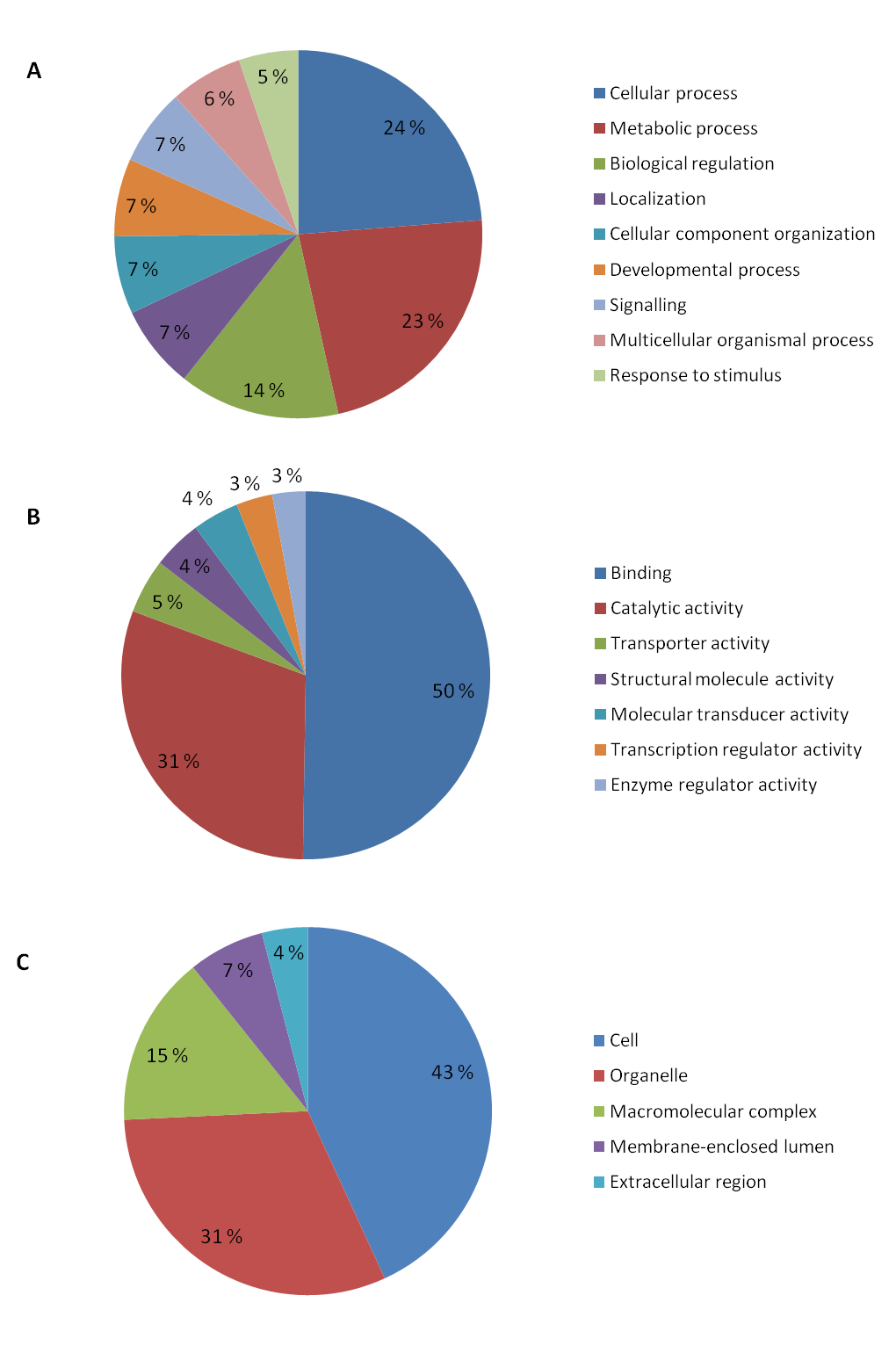

Supplement: Supplementary file 1 — Additional file 1: GO annotations for 10 k Atlantic halibut microarray. A: Biological processes, B: Molecular functions, C: Cell components. (DOCX 90 KB) [file 12864_2014_6689_MOESM1_ESM.docx]
