# Supplementary material for: Profiling of the embryonic Atlantic halibut (Hippoglossus hippoglossus L.) transcriptome reveals maternal transcripts as potential markers of embryo quality
Source: BMC Genomics. 2014 Sep 30;15(1):829. doi: 10.1186/1471-2164-15-829 (PMC4246526; doi:10.1186/1471-2164-15-829)
Supplement: Supplementary file 2 — Additional file 2: Biological process (BP) gene ontology annotations (GOs) for Atlantic halibut 10 k microarray probes. (DOCX 19 KB) [file 12864_2014_6689_MOESM2_ESM.docx]

Additional File 2 - Biological process (BP) gene ontology annotations (GOs) for Atlantic halibut 10k

microarray probes.

| **Level** | **GO ID** | **Term** | **#Seqs** | **Graph Score** |
| --- | --- | --- | --- | --- |
| 1 | GO:0008150 | biological process | 4060 | 5002 |
| 2 | GO:0009987 | cellular process | 2928 | 1676 |
| 2 | GO:0008152 | metabolic process | 2799 | 2542 |
| 3 | GO:0044238 | primary metabolic process | 2495 | 1541 |
| 3 | GO:0044237 | cellular metabolic process | 2030 | 899 |
| 2 | GO:0065007 | biological regulation | 1742 | 984 |
| 3 | GO:0050789 | regulation of biological process | 1709 | 1583 |
| 3 | GO:0043170 | macromolecule metabolic process | 1691 | 868 |
| 3 | GO:0009058 | biosynthetic process | 1432 | 850 |
| 4 | GO:0044260 | cellular macromolecule metabolic process | 1334 | 615 |
| 4 | GO:0034641 | cellular nitrogen compound metabolic process | 1176 | 504 |
| 3 | GO:0006807 | nitrogen compound metabolic process | 1176 | 302 |
| 4 | GO:0006139 | nucleobase, nucleoside, nucleotide and nucleic acid metabolic process | 1176 | 840 |
| 4 | GO:0019538 | protein metabolic process | 1143 | 778 |
| 2 | GO:0051179 | localization | 902 | 309 |
| 4 | GO:0006810 | transport | 902 | 859 |
| 3 | GO:0051234 | establishment of localization | 902 | 516 |
| 0 | GO:0071840 | cellular component organization or biogenesis | 844 | 366 |
| 2 | GO:0016043 | cellular component organization | 844 | 609 |
| 2 | GO:0032502 | developmental process | 841 | 764 |
| 4 | GO:0010467 | gene expression | 840 | 530 |
| 4 | GO:0044249 | cellular biosynthetic process | 832 | 311 |
| 5 | GO:0034645 | cellular macromolecule biosynthetic process | 832 | 518 |
| 4 | GO:0009059 | macromolecule biosynthetic process | 832 | 311 |
| 2 | GO:0023052 | signaling | 828 | 219 |
| 2 | GO:0032501 | multicellular organismal process | 787 | 500 |
| 3 | GO:0007275 | multicellular organismal development | 787 | 818 |
| 4 | GO:0023060 | signal transmission | 783 | 470 |
| 3 | GO:0023046 | signaling process | 783 | 282 |
| 4 | GO:0050794 | regulation of cellular process | 783 | 470 |
| 5 | GO:0007165 | signal transduction | 783 | 783 |
| 5 | GO:0044267 | cellular protein metabolic process | 762 | 467 |
| 5 | GO:0090304 | nucleic acid metabolic process | 713 | 470 |
| 3 | GO:0009056 | catabolic process | 698 | 698 |
| 2 | GO:0050896 | response to stimulus | 644 | 621 |
| 6 | GO:0006350 | transcription | 563 | 563 |
| 0 | GO:0071842 | cellular component organization at cellular level | 488 | 259 |
| 3 | GO:0006996 | organelle organization | 487 | 429 |
| 4 | GO:0043412 | macromolecule modification | 478 | 287 |
| 3 | GO:0006950 | response to stress | 478 | 478 |
| 6 | GO:0006464 | protein modification process | 478 | 478 |
| 3 | GO:0048869 | cellular developmental process | 406 | 244 |
| 4 | GO:0030154 | cell differentiation | 406 | 406 |
| 4 | GO:0006519 | cellular amino acid and derivative metabolic process | 380 | 380 |
| 3 | GO:0044281 | small molecule metabolic process | 380 | 228 |
| 3 | GO:0048856 | anatomical structure development | 353 | 212 |
| 4 | GO:0009653 | anatomical structure morphogenesis | 353 | 353 |
| 3 | GO:0007049 | cell cycle | 351 | 351 |
| 4 | GO:0006629 | lipid metabolic process | 302 | 302 |
| 5 | GO:0006811 | ion transport | 302 | 302 |
| 6 | GO:0006412 | translation | 301 | 301 |
| 5 | GO:0045184 | establishment of protein localization | 280 | 168 |
| 3 | GO:0033036 | macromolecule localization | 280 | 60 |
| 4 | GO:0008104 | protein localization | 280 | 101 |
| 5 | GO:0015031 | protein transport | 280 | 280 |
| 2 | GO:0016265 | death | 268 | 162 |
| 3 | GO:0008219 | cell death | 266 | 266 |
| 2 | GO:0008283 | cell proliferation | 259 | 259 |
| 4 | GO:0006091 | generation of precursor metabolites and energy | 253 | 253 |
| 4 | GO:0005975 | carbohydrate metabolic process | 250 | 250 |
| 6 | GO:0006259 | DNA metabolic process | 220 | 220 |
| 4 | GO:0009790 | embryo development | 220 | 220 |
| 3 | GO:0009605 | response to external stimulus | 207 | 207 |
| 4 | GO:0007010 | cytoskeleton organization | 192 | 192 |
| 3 | GO:0065008 | regulation of biological quality | 185 | 57 |
| 2 | GO:0000003 | reproduction | 178 | 178 |
| 3 | GO:0019725 | cellular homeostasis | 139 | 139 |
| 4 | GO:0042592 | homeostatic process | 139 | 83 |
| 2 | GO:0040007 | growth | 129 | 113 |
| 3 | GO:0007154 | cell communication | 126 | 99 |
| 3 | GO:0009719 | response to endogenous stimulus | 105 | 105 |
| 3 | GO:0007610 | behavior | 90 | 90 |
| 3 | GO:0009628 | response to abiotic stimulus | 84 | 84 |
| 3 | GO:0007267 | cell-cell signaling | 83 | 83 |
| 3 | GO:0009607 | response to biotic stimulus | 71 | 71 |
| 3 | GO:0019748 | secondary metabolic process | 67 | 67 |
| 7 | GO:0016049 | cell growth | 50 | 50 |
| 5 | GO:0032535 | regulation of cellular component size | 50 | 18 |
| 4 | GO:0090066 | regulation of anatomical structure size | 50 | 11 |
| 6 | GO:0008361 | regulation of cell size | 50 | 30 |
| 4 | GO:0007005 | mitochondrion organization | 45 | 45 |
| 4 | GO:0019222 | regulation of metabolic process | 33 | 7 |
| 6 | GO:0010468 | regulation of gene expression | 33 | 20 |
| 5 | GO:0060255 | regulation of macromolecule metabolic process | 33 | 12 |
| 7 | GO:0040029 | regulation of gene expression, epigenetic | 33 | 33 |
| 3 | GO:0008037 | cell recognition | 16 | 16 |
| 2 | GO:0016032 | viral reproduction | 15 | 15 |
| 2 | GO:0051704 | multi-organism process | 9 | 3 |
| 3 | GO:0044419 | interspecies interaction between organisms | 9 | 5 |
| 4 | GO:0044403 | symbiosis, encompassing mutualism through parasitism | 9 | 9 |
| 3 | GO:0007028 | cytoplasm organization | 3 | 3 |
