# Supplementary material for: Profiling of the embryonic Atlantic halibut (Hippoglossus hippoglossus L.) transcriptome reveals maternal transcripts as potential markers of embryo quality
Source: BMC Genomics. 2014 Sep 30;15(1):829. doi: 10.1186/1471-2164-15-829 (PMC4246526; doi:10.1186/1471-2164-15-829)
Supplement: Supplementary file 3 — Additional file 3: Molecular function (MF) gene ontology annotations (GOs) for Atlantic halibut 10 k microarray probes. (DOCX 16 KB) [file 12864_2014_6689_MOESM3_ESM.docx]

Additional File 3- Molecular function (MF) gene ontology annotations (GOs) for Atlantic halibut 10k

microarray probes.

| **Level** | **GO ID** | **Term** | **#Seqs** | **Graph Score** |
| --- | --- | --- | --- | --- |
| 1 | GO:0003674 | molecular function | 4391 | 3801 |
| 2 | GO:0005488 | binding | 3507 | 3447 |
| 3 | GO:0005515 | protein binding | 2221 | 2218 |
| 2 | GO:0003824 | catalytic activity | 2122 | 1422 |
| 3 | GO:0016787 | hydrolase activity | 911 | 732 |
| 3 | GO:0003676 | nucleic acid binding | 773 | 554 |
| 3 | GO:0000166 | nucleotide binding | 754 | 754 |
| 3 | GO:0016740 | transferase activity | 636 | 464 |
| 4 | GO:0003677 | DNA binding | 336 | 336 |
| 2 | GO:0005215 | transporter activity | 335 | 290 |
| 2 | GO:0005198 | structural molecule activity | 302 | 302 |
| 4 | GO:0003723 | RNA binding | 289 | 289 |
| 2 | GO:0060089 | molecular transducer activity | 286 | 127 |
| 3 | GO:0004871 | signal transducer activity | 286 | 211 |
| 5 | GO:0016301 | kinase activity | 259 | 198 |
| 4 | GO:0016772 | transferase activity, transferring phosphorus-containing groups | 259 | 119 |
| 4 | GO:0008233 | peptidase activity | 255 | 255 |
| 2 | GO:0030528 | transcription regulator activity | 222 | 222 |
| 2 | GO:0030234 | enzyme regulator activity | 205 | 205 |
| 4 | GO:0008092 | cytoskeletal protein binding | 200 | 190 |
| 5 | GO:0046872 | metal ion binding | 198 | 119 |
| 4 | GO:0043169 | cation binding | 198 | 71 |
| 3 | GO:0043167 | ion binding | 198 | 43 |
| 6 | GO:0005509 | calcium ion binding | 198 | 198 |
| 4 | GO:0004872 | receptor activity | 195 | 195 |
| 4 | GO:0005102 | receptor binding | 178 | 178 |
| 5 | GO:0016773 | phosphotransferase activity, alcohol group as acceptor | 156 | 94 |
| 6 | GO:0004672 | protein kinase activity | 156 | 156 |
| 3 | GO:0008289 | lipid binding | 134 | 134 |
| 5 | GO:0003779 | actin binding | 133 | 133 |
| 3 | GO:0003700 | sequence-specific DNA binding transcription factor activity | 128 | 128 |
| 2 | GO:0009055 | electron carrier activity | 115 | 115 |
| 4 | GO:0016788 | hydrolase activity, acting on ester bonds | 103 | 43 |
| 4 | GO:0008135 | translation factor activity, nucleic acid binding | 87 | 87 |
| 3 | GO:0030246 | carbohydrate binding | 80 | 80 |
| 5 | GO:0016818 | hydrolase activity, acting on acid anhydrides, in phosphorus-containing anhydrides | 64 | 14 |
| 4 | GO:0016817 | hydrolase activity, acting on acid anhydrides | 64 | 8 |
| 8 | GO:0003774 | motor activity | 64 | 64 |
| 6 | GO:0016462 | pyrophosphatase activity | 64 | 23 |
| 7 | GO:0017111 | nucleoside-triphosphatase activity | 64 | 38 |
| 3 | GO:0022857 | transmembrane transporter activity | 62 | 16 |
| 4 | GO:0022891 | substrate-specific transmembrane transporter activity | 56 | 20 |
| 5 | GO:0015075 | ion transmembrane transporter activity | 56 | 34 |
| 5 | GO:0015267 | channel activity | 56 | 20 |
| 3 | GO:0022892 | substrate-specific transporter activity | 56 | 12 |
| 4 | GO:0022803 | passive transmembrane transporter activity | 56 | 12 |
| 5 | GO:0022838 | substrate-specific channel activity | 56 | 34 |
| 6 | GO:0005216 | ion channel activity | 56 | 56 |
| 5 | GO:0004518 | nuclease activity | 53 | 53 |
| 5 | GO:0042578 | phosphoric ester hydrolase activity | 50 | 18 |
| 7 | GO:0004721 | phosphoprotein phosphatase activity | 50 | 50 |
| 6 | GO:0016791 | phosphatase activity | 50 | 30 |
| 3 | GO:0003682 | chromatin binding | 42 | 42 |
| 2 | GO:0016209 | antioxidant activity | 25 | 25 |
| 2 | GO:0045182 | translation regulator activity | 10 | 10 |
| 4 | GO:0005326 | neurotransmitter transporter activity | 6 | 6 |
| 3 | GO:0019825 | oxygen binding | 6 | 6 |
