# Supplementary material for: Profiling of the embryonic Atlantic halibut (Hippoglossus hippoglossus L.) transcriptome reveals maternal transcripts as potential markers of embryo quality
Source: BMC Genomics. 2014 Sep 30;15(1):829. doi: 10.1186/1471-2164-15-829 (PMC4246526; doi:10.1186/1471-2164-15-829)
Supplement: Supplementary file 4 — Additional file 4: Cellular component (CC) gene ontology annotations (GOs) for Atlantic halibut 10 k microarray probes. (DOCX 16 KB) [file 12864_2014_6689_MOESM4_ESM.docx]

Additional File 4 - Cellular component (CC) gene ontology annotations (GOs) for Atlantic halibut 10k

microarray probes.

| **Level** | **GO ID** | **Term** | **#Seqs** | **Graph Score** |
| --- | --- | --- | --- | --- |
| 1 | GO:0005575 | cellular component | 4090 | 2170 |
| 2 | GO:0005623 | cell | 3914 | 1779 |
| 3 | GO:0044464 | cell part | 3548 | 982 |
| 4 | GO:0005622 | intracellular | 3359 | 1300 |
| 4 | GO:0044424 | intracellular part | 3194 | 1453 |
| 2 | GO:0043226 | organelle | 2822 | 873 |
| 5 | GO:0043229 | intracellular organelle | 2762 | 1241 |
| 5 | GO:0005737 | cytoplasm | 2477 | 1732 |
| 3 | GO:0043227 | membrane-bounded organelle | 2371 | 891 |
| 6 | GO:0043231 | intracellular membrane-bounded organelle | 2371 | 1485 |
| 5 | GO:0044444 | cytoplasmic part | 1692 | 1303 |
| 7 | GO:0005634 | nucleus | 1420 | 1089 |
| 2 | GO:0032991 | macromolecular complex | 1362 | 788 |
| 3 | GO:0043234 | protein complex | 1164 | 1164 |
| 6 | GO:0043232 | intracellular non-membrane-bounded organelle | 943 | 607 |
| 3 | GO:0043228 | non-membrane-bounded organelle | 943 | 364 |
| 4 | GO:0044446 | intracellular organelle part | 737 | 311 |
| 3 | GO:0044422 | organelle part | 737 | 186 |
| 6 | GO:0044428 | nuclear part | 691 | 481 |
| 6 | GO:0070013 | intracellular organelle lumen | 608 | 245 |
| 4 | GO:0043233 | organelle lumen | 608 | 147 |
| 2 | GO:0031974 | membrane-enclosed lumen | 608 | 88 |
| 7 | GO:0031981 | nuclear lumen | 608 | 409 |
| 5 | GO:0005886 | plasma membrane | 543 | 543 |
| 4 | GO:0016020 | membrane | 543 | 326 |
| 7 | GO:0005739 | mitochondrion | 542 | 542 |
| 6 | GO:0005829 | cytosol | 519 | 519 |
| 6 | GO:0005654 | nucleoplasm | 482 | 482 |
| 7 | GO:0005856 | cytoskeleton | 421 | 409 |
| 2 | GO:0005576 | extracellular region | 366 | 248 |
| 7 | GO:0005783 | endoplasmic reticulum | 301 | 301 |
| 7 | GO:0005794 | Golgi apparatus | 266 | 266 |
| 6 | GO:0005840 | ribosome | 248 | 248 |
| 5 | GO:0030529 | ribonucleoprotein complex | 248 | 149 |
| 6 | GO:0005730 | nucleolus | 199 | 199 |
| 3 | GO:0044421 | extracellular region part | 174 | 93 |
| 7 | GO:0005694 | chromosome | 159 | 155 |
| 3 | GO:0031982 | vesicle | 142 | 51 |
| 7 | GO:0016023 | cytoplasmic membrane-bounded vesicle | 142 | 142 |
| 4 | GO:0031988 | membrane-bounded vesicle | 142 | 85 |
| 6 | GO:0031410 | cytoplasmic vesicle | 142 | 85 |
| 4 | GO:0005615 | extracellular space | 110 | 110 |
| 4 | GO:0031012 | extracellular matrix | 75 | 45 |
| 5 | GO:0005578 | proteinaceous extracellular matrix | 75 | 75 |
| 7 | GO:0005768 | endosome | 71 | 71 |
| 7 | GO:0005773 | vacuole | 69 | 37 |
| 4 | GO:0031975 | envelope | 67 | 24 |
| 4 | GO:0031967 | organelle envelope | 66 | 40 |
| 4 | GO:0012505 | endomembrane system | 66 | 40 |
| 5 | GO:0005635 | nuclear envelope | 66 | 66 |
| 7 | GO:0005815 | microtubule organizing center | 61 | 61 |
| 6 | GO:0044430 | cytoskeletal part | 61 | 37 |
| 8 | GO:0015630 | microtubule cytoskeleton | 61 | 37 |
| 7 | GO:0000323 | lytic vacuole | 56 | 34 |
| 8 | GO:0005764 | lysosome | 56 | 56 |
| 7 | GO:0000228 | nuclear chromosome | 55 | 55 |
| 7 | GO:0005777 | peroxisome | 38 | 38 |
| 7 | GO:0042579 | microbody | 38 | 23 |
| 6 | GO:0005811 | lipid particle | 13 | 13 |
| 5 | GO:0005929 | cilium | 12 | 12 |
| 4 | GO:0042995 | cell projection | 12 | 7 |
| 7 | GO:0009536 | plastid | 5 | 5 |
| 4 | GO:0030312 | external encapsulating structure | 3 | 3 |
| 5 | GO:0030313 | cell envelope | 1 | 1 |
| 5 | GO:0005618 | cell wall | 1 | 1 |
