# Supplementary material for: Profiling of the embryonic Atlantic halibut (Hippoglossus hippoglossus L.) transcriptome reveals maternal transcripts as potential markers of embryo quality
Source: BMC Genomics. 2014 Sep 30;15(1):829. doi: 10.1186/1471-2164-15-829 (PMC4246526; doi:10.1186/1471-2164-15-829)

Additional File 9 - Microarray validation.

Correlation plot of fold-change differences from 20 differentially expressed transcripts analyses by microarray and qPCR (*n* = 276). Correlation is given as Spearman’s Rho (ϱ). Abbreviations: H: High quality oocytes, L: Low quality oocytes; 8CS: 8-cell stage, GR: Germ ring, 10SS: 10-somite stage and HT: Hatched larvae.


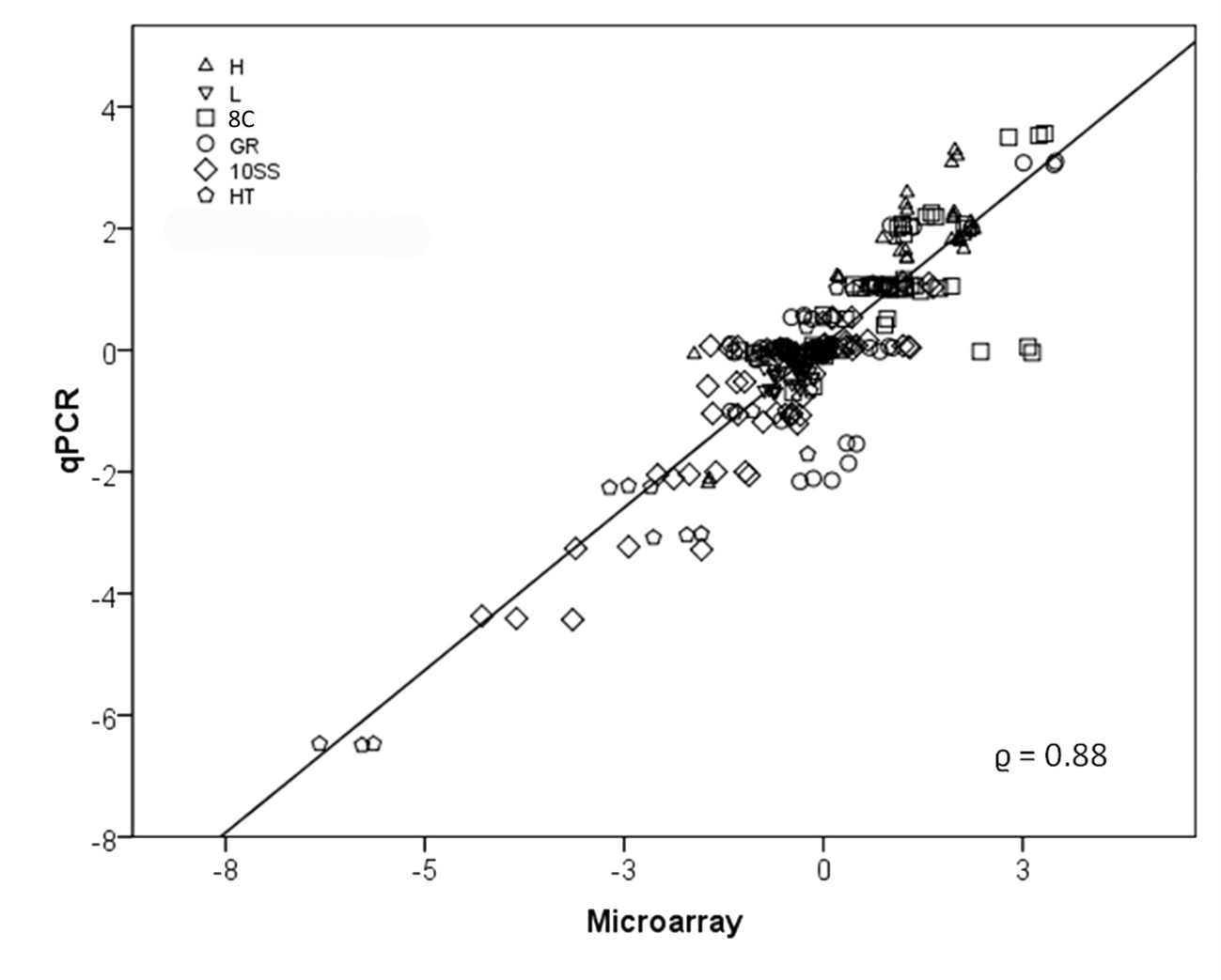

Supplement: Supplementary file 9 — Additional file 9: Microarray validation. Correlation plot of fold-change differences from 20 differentially expressed genes analyses by microarray and qPCR (n = 276). Correlation is given as Spearman’s Rho (ϱ). Abbreviations: H: High quality oocytes, L: Low quality oocytes; 8CS: 8-cell stage, GR: Germ ring, 10SS: 10-somite stage and HT: Hatched embryo. (DOCX 327 KB) [file 12864_2014_6689_MOESM9_ESM.docx]
