# Supplementary material for: Profiling of the embryonic Atlantic halibut (Hippoglossus hippoglossus L.) transcriptome reveals maternal transcripts as potential markers of embryo quality
Source: BMC Genomics. 2014 Sep 30;15(1):829. doi: 10.1186/1471-2164-15-829 (PMC4246526; doi:10.1186/1471-2164-15-829)
Supplement: Supplementary file 10 — Additional file 10: Primer information for microarray validation. (DOCX 18 KB) [file 12864_2014_6689_MOESM10_ESM.docx]

Additional File 10- Primer information for microarray validation.

| **Gene name (*Abbreviation*)** | **Accession number** | **Fwd (5ʹ🡒3ʹ)** | **Rev (5ʹ🡒3ʹ)** | **Size (bp)** | ***E* (%)** | ***R^2^*** |
| --- | --- | --- | --- | --- | --- | --- |
| Interferon regulatory factor 7 (*irf7*) | EB040633 | ACTGTCAATGTGGTTCCT | ATCGTAGAGGCTGTTGTG | 120 | 101 | 0.991 |
| Ring finger protein 213 (*rnf213*) | EB031933 | GATGGTCGTTGATCCTCAGTGT | CCTTGGCTACAGTGCTGGAA | 89 | 101 | 0.999 |
| Proteasome subunit beta type-9 precursos (*psmb9*) | EB041298 | AAATCTGGGATGTGAACG | TTTGATGAGGAAGAAGGTT | 113 | 100 | 0.999 |
| Mhc class 2A chain (*mhc2A*) | EB173954 | TGAGTAACTGGATGTGAATG | CTGGCATTGAGGTAGTTCTT | 165 | 99 | 0.992 |
| Programmed cell death 1 ligand 1 (*pd-1l*) | EB031478 | AGAAGGACGACTCAGACT | AACAGTGGCAGTGACATT | 127 | 104 | 0.993 |
| Membrane-spanning 4-domains subfamily a member 8a (*ms4a8a*) | EB040282 | GCCATAACAGCCATCGTA | TTAGGAGTATCAGAGTCAGAGT | 175 | 99 | 0.987 |
| Mhc class 1A chain (*mhc1A*) | EB036581 | TGATGTCTGGATGTGAATG | CTGGAGGAGGTAGTTCTT | 200 | 99 | 0.992 |
| Cytochrome p450 (*cyp2n*) | EB030401 | CATACCAAAGGGAACCTCTGT | GGTTGAAGGTGTCTGGAGTT | 86 | 98 | 0.992 |
| Eef1a2 binding protein (*eef1a2bp*) | EB038123 | GGGACGAGGAAAGTTGGA | ACATTGGCATATCAGCACATT | 84 | 97 | 0.987 |
| HH_90607090 | EB040531 | GGAGCAGATGGTATGATG | TTATGAGTGACGGTGTTG | 161 | 102 | 0.993 |
| HH_90603123 | EB036564 | ATGGTCACTATGGAGATTGT | CTCAGTTGTTAGCGGATAAG | 129 | 98 | 0.994 |
| HH_90988292 | EB103461 | TGTATCGTGGTGTTTCCTCTGT | ATTCACATCACTTCTGGGCAAAG | 177 | 97 | 0.997 |
| HH_90596347 | EB029788 | GGAGGAGGACTGGACATC | GCCACCAAGACAATCATCC | 134 | 98 | 0.997 |
| HH_193889799 | FK703165 | CAGCCCTACAGAAGAGTT | ATTGCTATTGAAGTTGGAACA | 200 | 100 | 0.997 |
| HH_166851124 | FD698747 | AACACAAGCCTCAGTCTC | TTCTGTCCATCCTTCATCC | 91 | 102 | 0.994 |
| HH_90606468 | EB039909 | TGGACGAAGGTTTCCTGTTG | GGTGTTGGCGATCAAGATGT | 86 | 100 | 0.987 |
| HH_Contig436 | EB036359 | GAAGCAGGTGGAACAATG | CACAGTTACCAGTCTTGATG | 125 | 100 | 0.993 |
| HH_90602035 | EB035476 | TCTCAAGACAGAGTTCCTCAAT | GAAGAAGACTGGAGCGTGTTA | 148 | 100 | 0.997 |
| HH_90599790 | EB033231 | GGCAGAAGAGCACAACATC | AACCTCCTGAATACTGTAGTGT | 200 | 103 | 0.995 |
| HH_Contig207 | EB041560 | GGAGTACAGCCAGATTCTATT | CTATGAATGACGGATGGAGTT | 178 | 100 | 0.999 |
| *β-Actin* (*Actβ*) | EB103323 | GAGAAGATGACTCAGATCATGTTCG | CCAGCCAGGTCCAGACGG | 154 | 89.0 | 0.999 |
| *β2-Tubulin* (*Tubb2*) | DT805564 | CTACAATGAGGCTTCAGGTGG | TCCCTCTGTGTAGTGACCCTTG | 134 | 93.9 | 0.998 |
| HHC00353 | CAAE00000000.1 | CGAGGTACTCTCCACTCTCATTCTC | AACCTCAGTTTTTATCCAGGTTCAC | 81 | 98 | 0.999 |
| HHC01517 | NM_001099229 | AGCAGGTTCTCCATGTTGAGTG | CTATTTCAAAGCCATGTTCACAGG | 143 | 95 | 0.998 |
| *Luciferase* (*Luc*) | M15077 | TCATTCTTCGCCAAAAGCACTCTG | AGCCCATATCCTTGTCGTATCCC | 198 | 99 | 0.995 |
|  |  |  |  |  |  |  |
