# Supplementary material for: Profiling of the embryonic Atlantic halibut (Hippoglossus hippoglossus L.) transcriptome reveals maternal transcripts as potential markers of embryo quality
Source: BMC Genomics. 2014 Sep 30;15(1):829. doi: 10.1186/1471-2164-15-829 (PMC4246526; doi:10.1186/1471-2164-15-829)

Additional File 11 - Raw cycle threshold (CT) levels (mean ± S.E.) of reference genes for qPCR normalization. A) *Actβ* and B) *Tubb2* in high (H) and low (L) quality Atlantic halibut oocytes (*n* = 8). C) *Luc* during early embryonic development of Atlantic halibut (*n* = 5). 8CS: 8-cell stage; GR: germ ring stage, 10SS: 10-somite stage, and HT: hatched larvae.


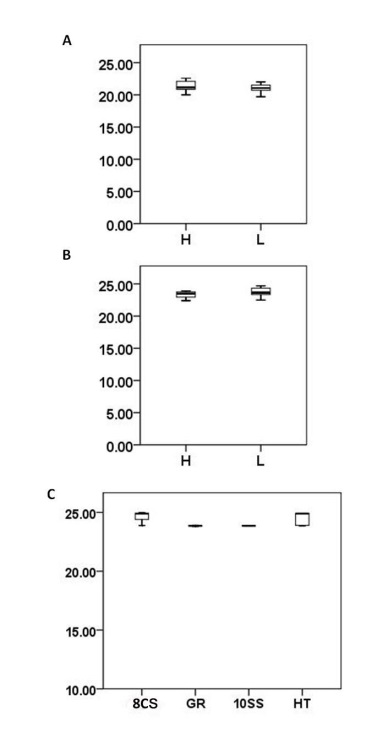

Supplement: Supplementary file 11 — Additional file 11: Raw cycle threshold (CT) levels (mean ± S.E.) of reference genes for qPCR normalization. A) Actβ and B) Tubb2 in high (H) and low (L) quality Atlantic halibut oocytes (n = 8). C) Luc during early embryonic development of Atlantic halibut (n = 5). 8CS: 8-cell stage; GR: germ ring stage, 10SS: 10-somite stage, and HT: hatched embryo. (DOCX 41 KB) [file 12864_2014_6689_MOESM11_ESM.docx]
